# Microbiome Datahub: an open-access platform integrating environmental metadata, taxonomy, and functional annotation for comprehensive metagenome-assembled genome datasets

**DOI:** 10.1186/s40168-026-02385-x

**Published:** 2026-03-16

**Authors:** Hiroshi Mori, Takatomo Fujisawa, Koichi Higashi, Yasuhiro Tanizawa, Zenichi Nakagawa, Hiroyo Nishide, Masaki Fujiyoshi, Yasukazu Nakamura, Ikuo Uchiyama, Motomu Matsui, Takuji Yamada

**Affiliations:** 1 Department of Informatics, National Institute of Genetics, 1111 Yata, Mishima, Shizuoka 411-8540 Japan; 2 Bioinformation and DDBJ Center, National Institute of Genetics, 1111 Yata, Mishima, Shizuoka 411-8540 Japan; 3 School of Life Science and Technology, Institute of Science Tokyo, Tokyo, 152-8550 Japan; 4 National Institute for Basic Biology, National Institutes of Natural Sciences, Okazaki, Japan; 5 Department of Integrated Biosciences, Graduate School of Frontier Sciences, The University of Tokyo, Kashiwa, Chiba 277-8561 Japan; 6 Institute for Chemical Research, Kyoto University, Uji, Kyoto 611-0011 Japan

**Keywords:** Metagenome, MAG, Database, Environment, Diversity

## Abstract

**Background:**

Metagenome-assembled genomes (MAGs) provide crucial insights into the genomic diversity of uncultured microbes. However, MAG datasets deposited in public repositories such as INSDC are often difficult to reuse due to heterogeneous quality, inconsistent taxonomic and functional annotations, and insufficiently curated environmental metadata. While secondary MAG databases such as MGnify, IMG/M, and SPIRE provide standardized resources, they reconstruct MAGs de novo from public metagenomic reads and therefore do not represent the original MAGs reported in publications.

**Results:**

To address this gap, we developed Microbiome Datahub, an open-access platform that systematically aggregates and re-annotates original MAGs from INSDC. We collected 214,427 MAGs, predicted genes by DFAST, performed quality assessment with CheckM, standardized taxonomic assignments with GTDB-Tk, inferred 27 phenotypic traits using Bac2Feature, assigned proteins to MBGD ortholog clusters and KEGG Orthology IDs using PZLAST, and annotated environmental metadata with the Metagenome and Microbes Environmental Ontology. Across these MAGs, the average completeness was 80.5% and contamination 1.8%; notably, the most frequent values were >95% completeness and <1% contamination, indicating that the majority of MAGs are of high quality. Comparative analyses showed that Microbiome Datahub provides phylogenetically and environmentally diverse MAGs: while the majority originated from vertebrate gut environments, a substantial number were also recovered from other habitats such as groundwater, including nearly 10,000 MAGs from the *Patescibacteria*. Inference of 27 phenotypic traits, including optimum growth temperature, further revealed ecological differentiation across phyla. Protein clustering revealed 56 million identity 40% clusters, with the majority unique compared with MGnify and GlobDB, and ~19% of proteins unassigned to MBGD ortholog clusters, underscoring their novelty.

**Conclusions:**

Microbiome Datahub integrates MAG genome sequences, gene and protein predictions, quality metrics, environmental and taxonomic annotations, ortholog cluster assignments, and phenotype predictions, all accessible via a web interface, API, and bulk downloads. By combining original MAGs with curated metadata and functional annotations, Microbiome Datahub constitutes a comprehensive and reusable resource that will accelerate microbiome and microbial genomics research.

Video Abstract

**Supplementary Information:**

The online version contains supplementary material available at 10.1186/s40168-026-02385-x.

## Background

Metagenome-Assembled Genome (MAG) analysis is a powerful approach that enables the recovery of genomic information from uncultured microbes directly from metagenomic sequencing data. It has become widely adopted in the field of environmental microbiology [1]. Although the publication of MAG datasets in scientific papers generally does not require mandatory MAG data deposition, the number of MAG sequences submitted to public repositories such as the International Nucleotide Sequence Database Collaboration (INSDC) has been rapidly increasing in recent years [2]. However, these publicly available MAG datasets are often difficult to reuse due to several challenges. The genome reconstruction methods vary across studies [3], resulting in inconsistent quality. Many MAGs lack standardized taxonomic or functional annotations, and crucial metadata such as habitat information, while optional in INSDC submissions, are not systematically organized, making it difficult to search or filter based on environmental context.

To address these issues, several large-scale secondary MAG databases (DBs) such as MGnify, IMG/M, and SPIRE have been developed by assembling publicly available metagenomic reads using uniform pipelines [4–6]. While these resources provide well-annotated and organized data, the reconstructed MAGs may differ significantly from those originally reported in publications due to differences in assembly and binning methodologies. Such differences include contig number, total genome size, and the exact contig sequences, which complicate the reproduction and evaluation of the original scientific findings.

At present, no comprehensive database exists that systematically aggregates, annotates, and organizes original MAGs as reported in the literature, covering a wide range of environments. To fill this gap, we developed Microbiome Datahub, an open-access platform that integrates MAG genome sequences with curated metadata and functional annotations. We collected over 210,000 MAG sequences and associated metadata from INSDC repositories. Each MAG was subjected to quality assessment, standardized taxonomic assignment, and gene annotation. Environmental metadata were curated using the Metagenome and Microbes Environmental Ontology (MEO) [7], enabling systematic cross-environment comparisons. Furthermore, 27 phenotypic traits were predicted with Bac2Feature [8], and MAG-derived proteins were assigned to MBGD ortholog clusters and KEGG Orthology IDs using a high-speed sequence similarity search [9, 10]. To explore protein novelty, we also performed large-scale clustering of the MAG-derived proteins and compared them with protein resources from MGnify and GlobDB [11, 12].

The resulting platform, Microbiome Datahub, provides curated, searchable, and reusable MAG resources encompassing genome sequences, gene and protein predictions, quality metrics, environmental and taxonomic annotations, ortholog cluster assignments, and phenotype predictions. By integrating diverse prokaryotic datasets with rich contextual annotations and providing programmatic as well as bulk access, Microbiome Datahub constitutes a comprehensive and sustainable resource that will accelerate microbiome and microbial genomics research.

## Materials and methods

### Data collection

MAG data were retrieved from NCBI Datasets in May 2023 [13]. We collected MAG assemblies registered under the INSDC identifiers (GCA ID), rather than those in the RefSeq DB. To obtain associated metadata for each MAG, we downloaded the assembly_summary_genbank.txt file from the ASSEMBLY REPORTS DB [14], as well as BioProject and BioSample metadata in XML format from the NCBI BioProject and BioSample DBs [15, 16].

### Metadata annotation

The workflow for metadata extraction is shown in Additional File 1. Basic metadata for each MAG were obtained from the ASSEMBLY REPORTS TSV file using the corresponding GCA accession. Additional metadata were extracted from the BioSample XML files by mapping each GCA ID to its associated BioSample ID. More than 99% of the MAGs were linked to dedicated BioSample IDs that differed from those associated with the original metagenomic read datasets used to construct the MAGs. These MAG-specific BioSamples (hereafter referred to as MAG-BioSamples) sometimes lacked key environmental descriptors, such as the isolation source, necessitating additional metadata retrieval. For metagenomes, the NCBI Taxonomy name often includes environmental descriptors such as “soil metagenome” or “human gut metagenome” [17]. Accordingly, we also targeted the OrganismName attribute in BioProject metadata and the Organism taxonomy name attribute in BioSample metadata for environmental information. Environmental descriptors were extracted from the following fields: BioProject: OrganismName, Name, Title; MAG-BioSample: Title, OrganismName, Comment, name, host, biome, feature, material, env_package, isolation-source; Derived-from BioSample: Title, Organism, Comment, host, biome, feature, material, env_package, isolation-source.

All extracted environmental metadata were first automatically annotated using the Metagenome and Microbes Environmental Ontology (MEO), a microbial habitat ontology developed by us [18]. The annotations were then manually curated, and up to two MEO IDs were assigned to each MAG. In addition, the host NCBI Taxonomy ID was also annotated where applicable.

### Genome annotation

**Fig. 1:**
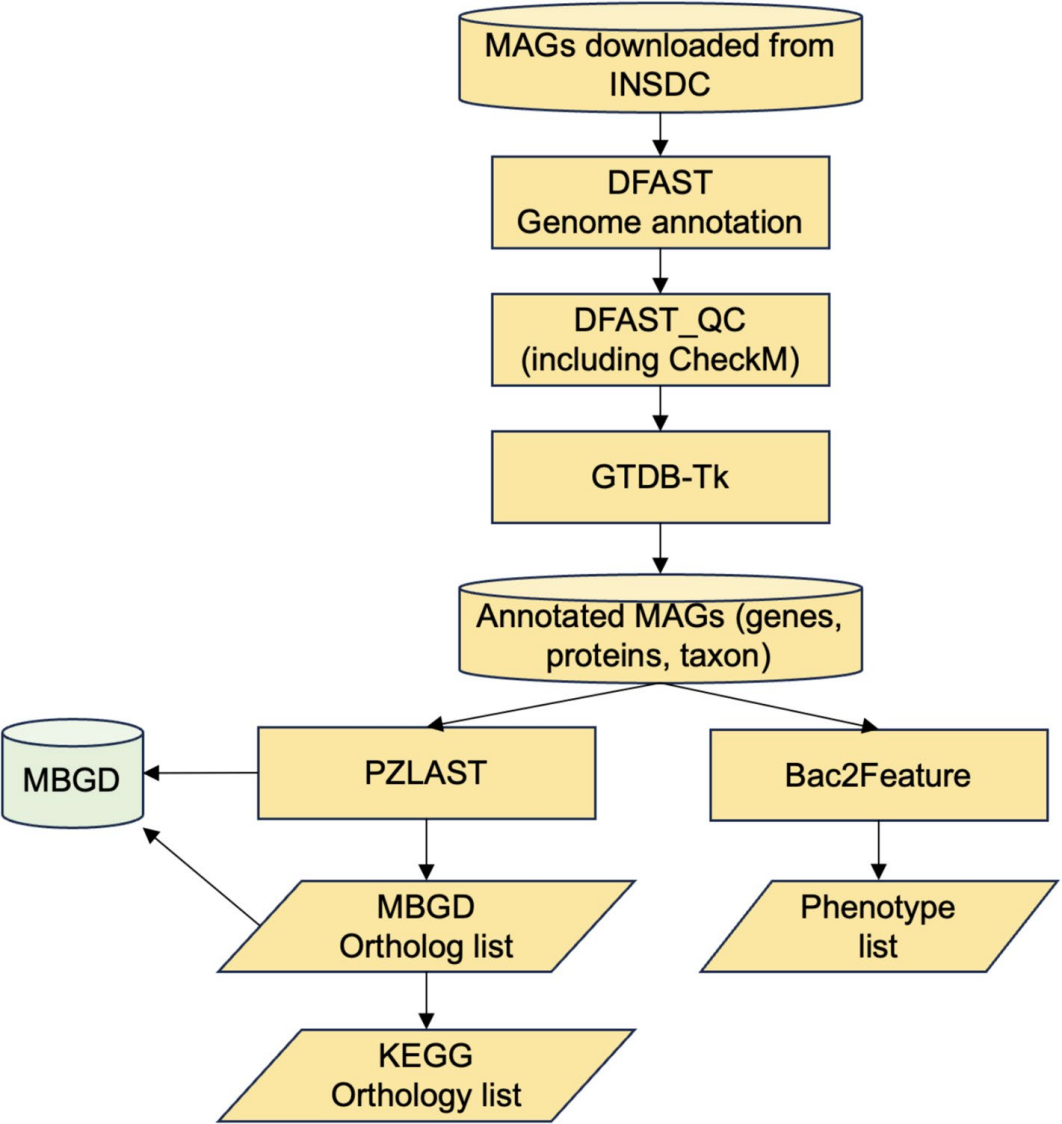
MAG sequence annotation workflow. Rather than reassembling or reconstructing MAGs from INSDC data, we re-annotated the original MAG sequences deposited in INSDC

Genome annotation of each MAG was performed following the workflow illustrated in Fig. 1. Gene prediction was conducted using DFAST, an automated annotation pipeline for prokaryotic genomes [19]. Genome quality was assessed with DFAST_QC [20], which incorporates CheckM for evaluating completeness and contamination [21]. Taxonomic assignment was performed using GTDB-Tk [22], with GTDB release 220 as the reference database [23]. Based on the resulting taxonomic assignments, 27 phenotypic traits were predicted for each MAG using Bac2Feature [8]. In this study, phenotype inference was performed based on taxonomic names, rather than 16S rRNA gene sequences, because most MAGs lack 16S rRNA genes. The resulting phenotype predictions should therefore be interpreted as typical or consensus phenotypes associated with a given taxon, rather than as direct evidence derived from the genomic content of individual MAGs. The predicted phenotypes included: cell diameter (log 10 µm), cell length (log 10 µm), doubling time (log10 hours), growth temperature (°C), optimum growth temperature (°C), optimum pH, genome size (bp), GC content (%), number of coding genes, number of 16S rRNA genes, number of tRNA genes, Gram stain (probability: 0–1), sporulation (probability: 0–1), motility (probability: 0–1), salinity (probability: 0–1), respiration type (facultative, anaerobic, aerobic; probability: 0–1), temperature range (mesophilic, thermophilic, psychrophilic; probability: 0–1), and cell shape (bacillus, coccus, filament, coccobacillus, vibrio, spiral; probability: 0–1). Phenotype prediction was attempted sequentially at the species, genus, and family levels; once a phenotype could be assigned at a given taxonomic level, prediction at higher ranks was not performed. If a phenotype could not be predicted even at the family level, the value for that category was recorded as NA.

All predicted protein sequences from each MAG were searched against 12,093,450 protein sequences derived from orthologous clusters of representative isolate genomes in the Microbial Genome Database for Comparative Analysis (MBGD) [9], considering only clusters with at least three member sequences. The sequence similarity searches were performed using customized PZLAST [24], a high-speed sequence similarity search tool optimized for metagenomic data, with the parameter setting lMer = 6 and 32,768 thread parallelization on the PEZY-SC3 processor [25]. The top hit with an E-value < 1e-8 was used to assign the corresponding MBGD ortholog cluster ID to each MAG protein. Since MBGD ortholog clusters are linked to KEGG Orthology (KO) IDs, the corresponding KO ID was assigned to each MAG protein based on its matched MBGD ortholog cluster [10]. The KEGG Module [10] composition of each MAG was then calculated using Genomaple [9], with module completion ratios computed from KO composition. Modules with a completion ratio ≥ 80% and q-value ≤ 0.25 were considered present.

To assess de novo protein sequence diversity in Microbiome Datahub, protein sequences were clustered using the Linclust algorithm implemented in MMseqs2 (version 18-8cc5c) at 90% and 40% sequence identity [26]. The resulting sequence clusters were compared with 90% identity sequence clusters from MGnify and with sequence clusters (size > 1) from GlobDB [11, 12]. In addition, protein sequences from MGnify and GlobDB were independently clustered at 40% sequence identity to facilitate direct comparisons across resources. All clustering procedures were applied using consistent parameters to protein datasets from the three resources, enabling a systematic comparison of cluster composition and overlap.

### Database construction

Genome statistics, metadata, phenotype predictions from Bac2Feature, and MBGD ortholog cluster assignment results for each MAG were converted into JSON format and indexed in Elasticsearch [27], enabling efficient filtering and search based on various metadata fields. For each MAG, the following data were made available for download via an Application Programming Interface (API): genome sequences, gene nucleotide sequences, predicted protein sequences, assigned MBGD ortholog cluster IDs, associated KO IDs, KEGG Module composition, predicted phenotypes, environmental metadata including MEO annotation, and taxonomic annotations. The web user interface was developed using React [28], allowing users to search, browse, and download MAG data through a web browser. The resulting DB, Microbiome Datahub, provides a user-friendly interface for accessing and exploring comprehensive MAG datasets [29].

### Database comparison

For comparative analyses, statistics from other large MAG databases (MGnify, IMG/M, and SPIRE) were obtained from the latest publicly available releases of each database at the time of analysis (December 2025). Statistics for MGnify were collected from the Genome Catalogues list page (https://www.ebi.ac.uk/metagenomics/browse/genomes) and the peptide database README file (https://ftp.ebi.ac.uk/pub/databases/metagenomics/peptide_database/current_release/README.txt). Statistics for SPIRE were obtained from the SPIRE Downloads page (https://spire.embl.de/downloads). Statistics for IMG/M were obtained from the Metagenome Bins information pages (https://img.jgi.doe.gov/cgi-bin/m/main.cgi?section=MetagenomeBins&page=info; https://img.jgi.doe.gov/cgi-bin/m/main.cgi?section=MetagenomeBins&page=bins_by_phylogenetic_category&taxonomy=gtdbtk&category=domain).

To evaluate the impact of different assembly and binning pipelines on MAG properties, we performed a targeted comparative analysis between original MAGs deposited in INSDC and curated in Microbiome Datahub, and MAGs reconstructed by secondary databases. Twenty representative MAGs spanning 15 phyla and diverse environments were selected from Microbiome Datahub. For each selected MAG, corresponding MAGs in MGnify and SPIRE were searched based on shared BioProject and BioSample identifiers, taxonomic assignment, and sequence similarity. Average nucleotide identity (ANI) was calculated by using OrthoANIu to confirm genomic correspondence [30]. Genome statistics, including completeness, contamination, genome size, and contig number, were retrieved from each database and compared across resources. IMG/M was not included in this comparison because it primarily hosts MAGs generated from Joint Genomics Institute’s sequencing projects [5], and relatively few MAGs reconstructed from INSDC-deposited metagenomic reads were available for direct comparison.

### Identification of environment-specific KEGG Modules independent of phylogenetic bias

To identify KEGG Modules that are specifically associated with particular environments while minimizing the influence of phylogenetic composition, we conducted a multi-step statistical analysis. For each environment–module pair (i.e., MEO class–KEGG Module pair), we first calculated the environment-specific module frequency across MAGs. This frequency was compared against the corresponding frequency in all other environments combined. Environment–module pairs showing enrichment were identified using a 2  ×  2 contingency table and Fisher’s exact test, followed by Benjamini–Hochberg false discovery rate (FDR) correction. Only pairs exceeding minimum thresholds for absolute MAG counts (>30) and relative module frequency (>20% of MAGs carrying the module within the environment) were retained as candidate environment-enriched modules.

To distinguish true environment-specific functional signals from those driven by uneven phylogenetic distributions, we further evaluated candidate environment–module pairs while controlling for phylum-level composition. For each candidate pair, we constructed stratified 2 × 2 contingency tables across phyla, comparing module presence and absence across MAGs between the target environment and all other environments within each phylum. These stratified tables were analyzed using the Cochran–Mantel–Haenszel (CMH) test, which estimates a common odds ratio while accounting for phylum as a stratification variable [31]. The Mantel–Haenszel common odds ratio was used as a measure of effect size [32], and CMH test* p*-values were adjusted for multiple testing using the Benjamini–Hochberg procedure. To further ensure robustness, we required that environment enrichment be supported across multiple phyla. Specifically, a module–environment association was retained only if enrichment in the target environment was observed in the same direction (odds ratio > 1) in at least three independent phyla with sufficient MAG numbers. This criterion reduces the likelihood that observed enrichment is driven by a small number of dominant phyla.

KEGG Modules were classified as environment-specific if they satisfied all of the following criteria: (i) significant enrichment in the target environment relative to other environments (FDR-adjusted *p*-value < 0.05), (ii) a Mantel–Haenszel odds ratio indicating substantial effect size after phylum stratification, and (iii) consistent enrichment across multiple phyla. KEGG Modules failing these criteria were considered likely to reflect phylogenetic composition.

## Results

### Microbiome Datahub overall statistics

We collected 214,427 MAGs from INSDC, which were subjected to quality evaluation, gene annotation, taxonomic assignment, environmental annotation, phenotype prediction, and ortholog cluster assignment. A comparison of basic statistics with other large-scale MAG databases is provided in Table 1. The compared MAG databases apply different inclusion criteria. Microbiome Datahub and SPIRE do not impose completeness or contamination thresholds for MAG inclusion. In contrast, IMG/M includes only medium- and high-quality MAGs following the Minimum Information about a MAG (MIMAG) definition [2], requiring a minimum completeness >50% and contamination <10%. MGnify requires a contamination threshold <5% and a quality score ≥ 50, calculated as completeness (%) – 5 × contamination (%), which effectively corresponds to a completeness threshold of approximately >50% depending on contamination levels [11]. These differences in quality filtering policies likely contribute to the observed variation in the number of MAGs and predicted proteins across databases. Definitions of environmental categories differ among databases: Microbiome Datahub uses MEO; MGnify employs only 18 in-house–defined biomes [4]; IMG/M uses the GOLD Ecosystem classification [5]; and SPIRE relies on its own microntology system [6]. Taxonomic classifications also differ among databases: IMG/M uses GTDB release r226, Microbiome Datahub uses GTDB release r220, whereas SPIRE and a subset of MGnify entries are based on GTDB release r207. Project definitions are likewise database-specific: Microbiome Datahub and SPIRE are organized primarily by INSDC BioProject, MGnify by ENA Study/Project, and IMG/M by GOLD Study. The metric “average MAGs per project” indicates that SPIRE mainly covers large-scale projects, while the limited number of environments in MGnify suggests a focus on specific environments such as the gut. Interestingly, although MGnify contains fewer than half the number of MAGs and total bases compared with SPIRE, it reports a higher number of proteins. Similarly, despite having approximately twice the total bases of Microbiome Datahub, MGnify reports nearly six times as many proteins, likely because it includes predictions from both FragGeneScan and Prodigal [4, 33, 34].

**Table 1:** Basic statistics of major MAG Databases

	Microbiome Datahub	MGnify	IMG/M	SPIRE
Number of MAGs	214,427	518,533	268,973	1,158,553
Number of environments	123	18	178	111
Number of phyla	213	182	197	182
Number of proteins	454,799,231	2,455,939,992	791,399,378	2,452,036,556
Total nucleotides (bp)	533,632,659,876	1,291,075,397,313	784,800,237,694	2,627,577,000,000
Number of projects	1,759	5,189	8,929	739
Average completeness (%)	80.59	87.07	76.89	81
Average contamination (%)	1.83	1.03	2.10	1.77
Average MAGs per project	121.9	99.9	30.1	1567.7
Minimum completeness (%)	N.A	50	50	N.A
Minimum contamination (%)	N.A	5	10	N.A
Last data update date	May, 2023	Nov, 2025	Dec, 2025	Sep, 2023

Across the four databases, the average MAG completeness (~76%) and contamination (~3%) are consistent, suggesting a shared consensus on quality thresholds when selecting which MAGs to use. In Microbiome Datahub, 172,716 MAGs with CheckM completeness >60% and contamination <10% were retained for downstream analyses in this study, applying a more stringent completeness criterion than the MIMAG standard (>50%) to support robust metabolic module analyses. The distribution of completeness and contamination is shown in Additional File 2. The mean completeness was 80.5% and mean contamination was 1.8%, but the most frequent values were notably higher: >95% completeness and <1% contamination. These results indicate that most studies deposit only high-quality MAGs into public repositories.

The distributions of contig number and genome size are summarized in Additional File 3. The mean contig number per MAG was 222, and the mean genome size was 2,594,757 bp. More than 99% of projects used short-read sequencing, and the number of MAGs reconstructed as complete genomes with a single contig was extremely small (<1000). This number is expected to increase with applying the long-read sequencing. For IMG/M, the mean contig number per MAG was 266, and the mean genome size was 2,917,765 bp. These statistics are consistent with those observed in Microbiome Datahub. For comparison, 13,174 RefSeq draft genomes of type strains sequenced by short-read platforms after 2010 had a mean of 71 contigs [13], indicating that MAGs generally have much higher fragmentation. This difference reflects the increased complexity of metagenomic assemblies. Indeed, the distribution of average contig length for MAGs is shown in Additional File 4, with a median of 15,722 bp. While this length exceeds the minimum contig thresholds required by most binning tools (several kb) [3], it is approximately one-quarter of the average contig length (66 kb) in RefSeq draft genomes sequenced after 2010, again highlighting the greater difficulty of assembling metagenomes compared to isolate genomes.

As previously noted in many studies, MAG reconstruction relies on clustering contigs based on tetranucleotide frequencies and other metrics, which often results in the exclusion of specific regions (e.g., rRNA genes) [35, 36]. Consistent with this, 88% of MAGs in Microbiome Datahub lack 16S rRNA gene sequences. This highlights a current limitation of short-read MAGs: even when 16S rRNA gene amplicon sequencing and shotgun metagenome sequencing are performed on the same sample, it remains challenging to directly integrate MAG data with 16S rRNA gene amplicon results.

### Comparison of original MAGs with re-assembled MAGs in secondary databases

To empirically assess the impact of different assembly and binning pipelines on MAG characteristics, we performed a targeted comparison between original MAGs deposited in INSDC and curated in Microbiome Datahub, and MAGs reconstructed from the same BioProject and BioSample in secondary databases, including MGnify and SPIRE (Additional File 5). We selected 20 representative MAGs spanning 15 phyla and diverse environments from Microbiome Datahub. For each MAG, corresponding MAGs in MGnify and SPIRE were identified based on shared BioProject and BioSample information, consistent taxonomic assignment, and high sequence similarity. ANI was calculated to confirm genomic correspondence.

In MGnify, some MAGs were identical to original INSDC submissions; however, the majority were re-assembled from metagenomic reads. Even when closely related MAGs were recovered (often with ANI > 99%), notable differences were observed in genome statistics, including genome size, completeness, contamination, and contig number. In SPIRE, all matched MAGs were reconstructed de novo from metagenomic reads, and in several cases, no corresponding MAG could be identified despite the presence of the same BioProject and BioSample in the database. For matched SPIRE MAGs, contig numbers and genome sizes consistently differed from those of the original INSDC MAGs.

These results indicate that assembly and binning strategies can substantially influence the resulting MAG properties, even when based on identical sequencing data. This provides direct empirical support for the importance of curating and re-annotating original MAGs as reported in the literature, as implemented in Microbiome Datahub.

### MAG taxonomic distribution

**Fig. 2:**
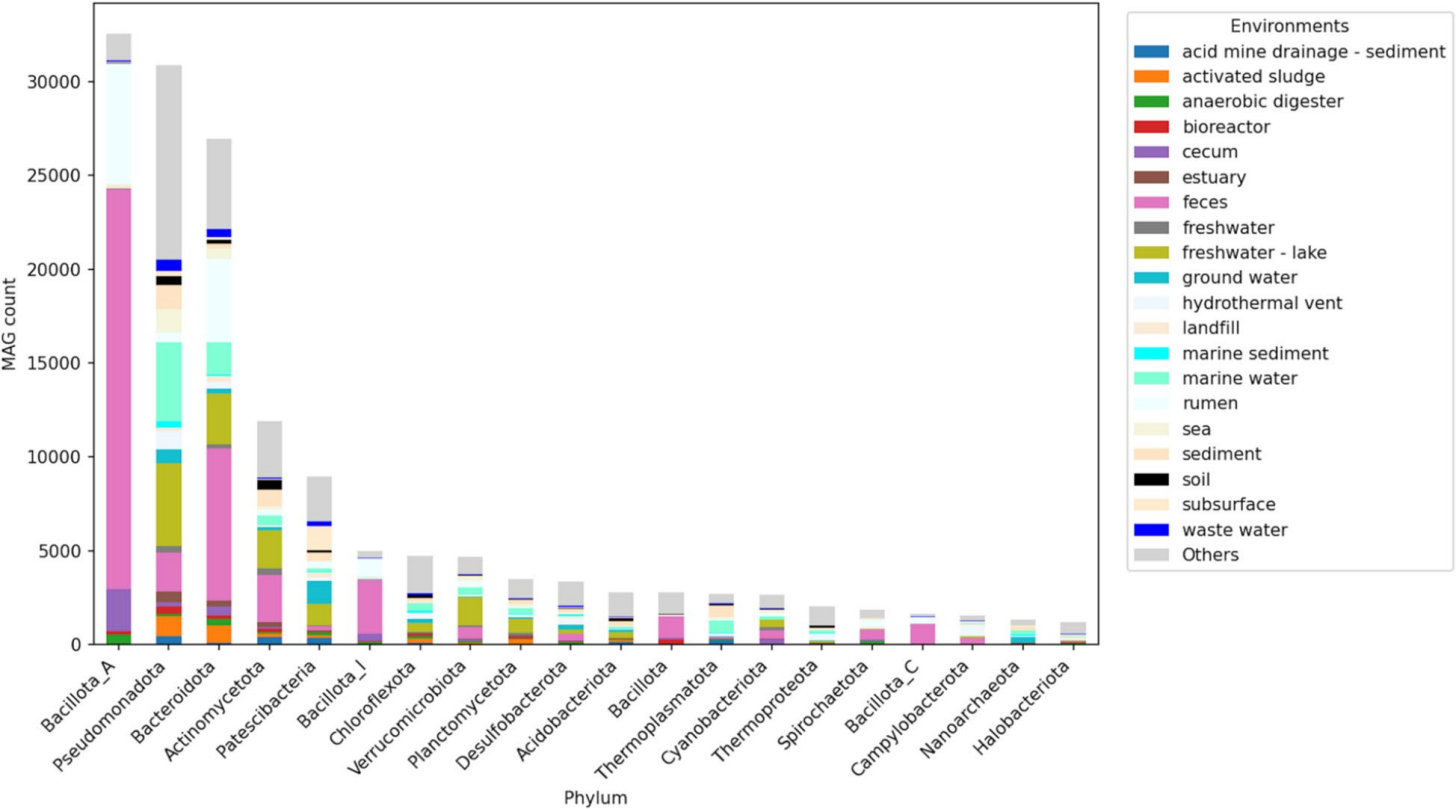
Bar plot of the phylogenetic distribution and environmental distribution of Microbiome Datahub MAGs. The top 20 archaeal and bacterial phyla are shown according to the number of MAGs. The 20 most abundant MEO environment classes are shown in distinct colors. All other environments are grouped into a single category, shown in gray

The phylogenetic distribution of MAGs in Microbiome Datahub is summarized in Fig. 2. The most abundant phyla include *Bacillota*_A, *Pseudomonadota*, *Bacteroidota*, and *Actinomycetota*, which are also abundant among isolate genomes and contain many human gut microbes [37]. In addition, nearly 10,000 MAGs were recovered from the *Patescibacteria*, a phylum that is difficult to culture, characterized by reduced genome sizes, and thought to parasitize other bacteria [38].

**Fig. 3:**
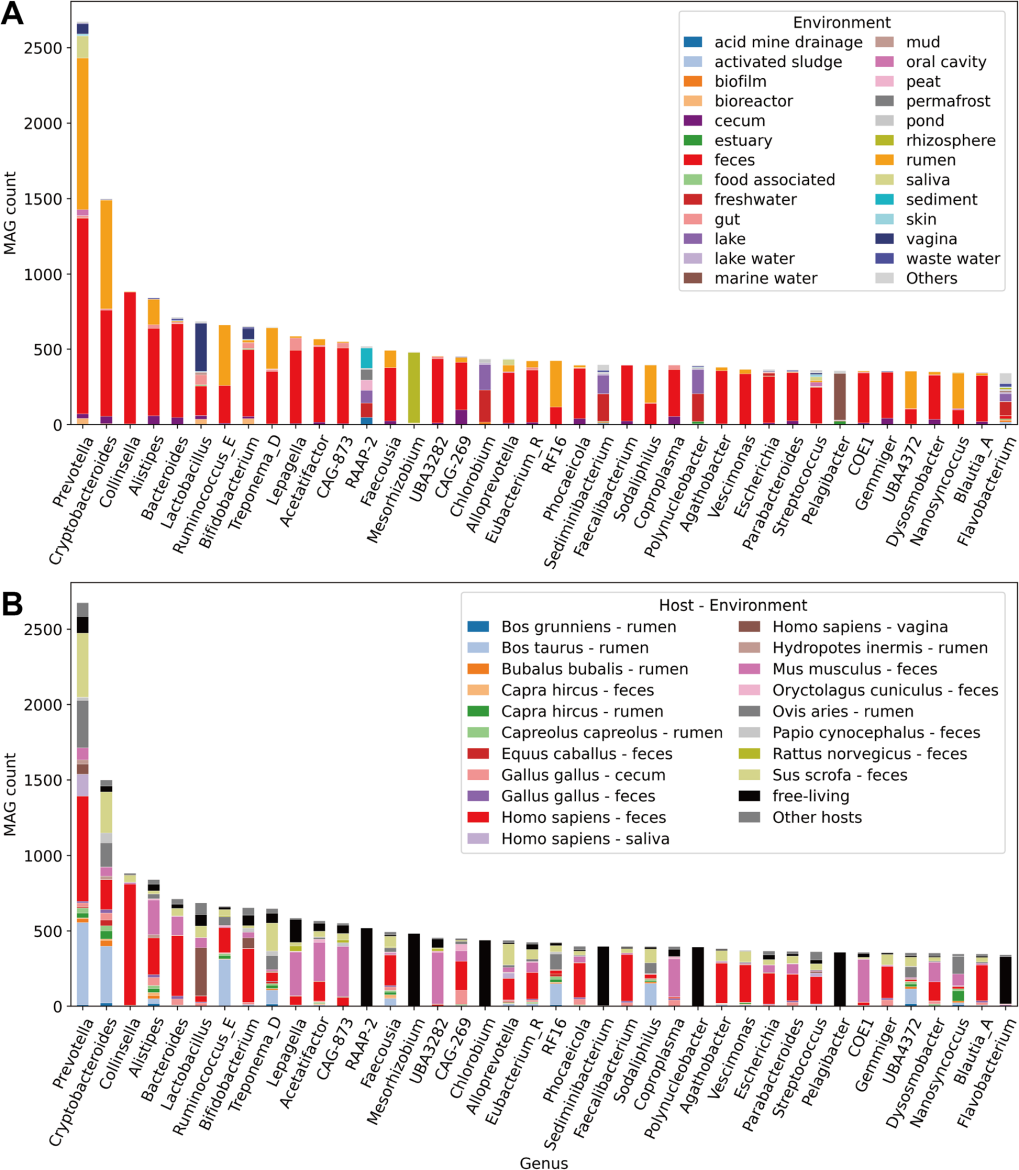
**A **Environmental distribution of the 40 most abundant genera. The 25 most abundant MEO environment classes are shown in distinct colors, while all other environments are grouped into a single category shown in gray. **B **Host–environment pair distribution of the 40 most abundant genera. The 19 most abundant MEO environment class–host taxonomic name pairs are shown in distinct colors. MAGs from free-living environments are shown in black. All other environment–host pairs (excluding free-living) are grouped into a single category shown in dark gray. Red indicates human feces, and pink indicates mouse feces

The environmental distribution of the top 20 phyla is also summarized in Fig. 2. All phyla were derived from multiple environments, with no phylum restricted to a single habitat. For the top 20 phyla, each was detected in at least five distinct MEO environment classes. For example, although *Patescibacteria* are often associated with oligotrophic environments [38], one order within this phylum—*Saccharimonadales* (formerly TM7)—is also known to inhabit the human oral and gut [39]. In contrast, most members of *Bacillota*_A and *Bacillota*_I were enriched in vertebrate intestinal environments such as feces, rumen, and cecum. At a finer taxonomic resolution, the environmental distribution of the 40 most abundant genera is shown in Fig. 3A. Except for genera such as *Flavobacterium*, the majority of the 40 most abundant genera showed clear habitat preference, with more than 80% of these genera originating from one or two dominant environments. Notably, genera predominantly associated with feces and rumen environments accounted for more than half of the 40 most abundant genera represented in Microbiome Datahub MAGs (i.e., abundant in the public repository). Focusing on host-associated MAGs, the correspondence between host species and environments for these 40 genera is presented in Fig. 3B. This analysis revealed genera such as UBA3282, *Coproplasma*, COE1, UBA4372, and *Nanosyncoccus*, which are rarely detected in the human gut but frequently recovered from the guts or rumen of other vertebrates.

Genome size distributions for MAGs belonging to the 30 most represented phyla are summarized in Additional File 6. As previously reported, *Patescibacteria*, *Nanoarchaeota*, and *Micrarchaeota* had consistently small genomes (median < 1 Mb) [40], whereas *Myxococcota* displayed a wide range of genome sizes but with a median exceeding 5.6 Mb [41]. These data suggest that genome size distributions differed substantially among phyla, indicating phylum-level ecological differentiation. The phylum-level distributions of MAGs across the 20 most common environments are shown in Additional File 7. In certain environments, such as groundwater and subsurface habitats, MAGs were strongly dominated by *Patescibacteria*. However, most environments comprised diverse compositions of phyla.

### MAG phenotype distribution among environment

**Fig. 4:**
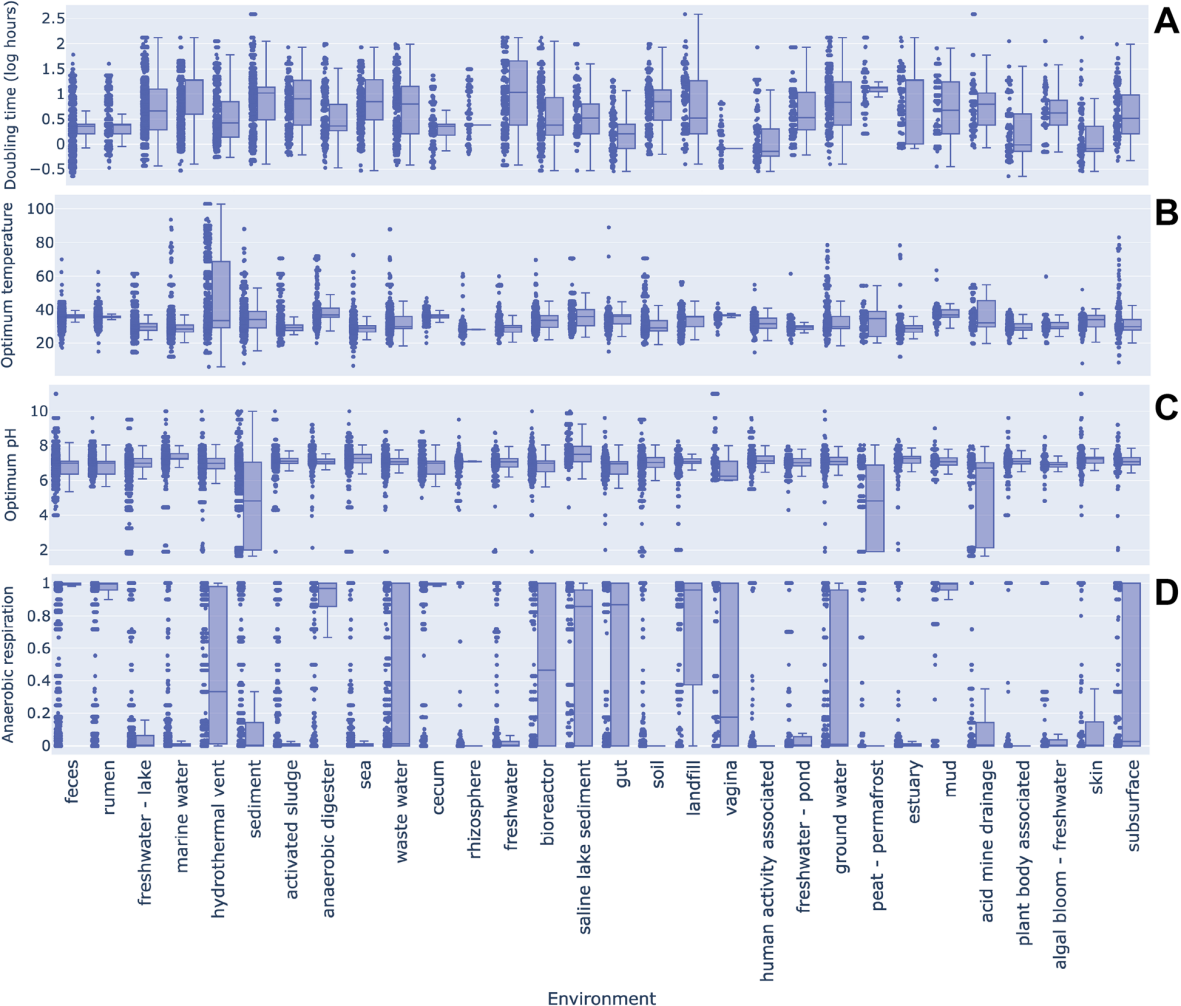
Phenotypic trait predictions for MAGs from the 30 most abundant environments using Bac2Feature. **A **Per-phylum boxplots of predicted doubling times; the y-axis represents the natural logarithm of doubling hours. **B **Per-phylum boxplots of predicted optimum growth temperatures.** C **Per-phylum boxplots of predicted optimum pH values.** D **Per-phylum boxplots of predicted anaerobic respiration potentials; the y-axis represents the predicted potential of each MAG taxon to perform anaerobic respiration. In all panels, boxes indicate the interquartile range with median values shown as horizontal lines, whiskers represent 1.5× the interquartile range, and points represent individual MAGs. These plots are intended to summarize overall distributional trends across environments rather than to highlight individual data points

Phenotypic traits of MAGs were inferred using Bac2Feature, which predicts 27 phenotypes such as doubling time and optimum growth temperature based on taxonomic name. Among the 172,716 high-quality MAGs (completeness > 60%, contamination < 10%), 52.8% could be assigned at least one phenotype. Detailed distributions of phenotypes across taxa are described in the original Bac2Feature publication [8]. Representative results are shown in Fig. 4, which summarizes predictions for doubling time, optimum growth temperature, optimum pH, and anaerobic respiration potential across MAGs from the 30 most abundant environments. MAGs from host-associated environments such as feces, rumen, cecum, rhizosphere, vagina, plant-body associated, and skin exhibited shorter median doubling times compared with MAGs from free-living environments (e.g., soil, marine water, and freshwater) (Fig. 4A), indicating distinct microbial strategies in host-associated habitats [42]. For optimum growth temperature (Fig. 4B), MAGs from hydrothermal vents displayed high variance, consistent with the coexistence of hyperthermophiles and mesophiles depending on distance from vent emissions [43]. Optimum pH predictions showed large variance in acid mine drainage, peat, and sediment samples, reflecting the acidic conditions characteristic of these habitats (Fig. 4C). Anaerobic respiration potentials ranged from 0 to 1, with higher values indicating stronger possibility for anaerobic respiration (Fig. 4D). Anaerobic environments such as gut-associated habitats, anaerobic digesters, and landfills showed high anaerobic respiration potentials, with median values >0.85, whereas free-living environments displayed broader distributions with lower medians. In contrast, environments with wide distributions likely reflect mixtures of both anaerobic and non-anaerobic samples (e.g., bioreactor).

### MAG protein diversity

MAGs provide access to complete protein sequences from uncultured microbes, which are critical for exploring protein diversity and are widely used in applications including protein structure prediction [44]. At present, metagenome-derived protein sequences from MGnify are used as reference datasets for AlphaFold 2/3 [45, 46]. Similarly, IMG/M has been used for analyses of novel protein families from metagenomic proteins [44]. MGnify also offers a HMMER-based search service for proteins derived from MAGs [11]. We previously developed PZLAST, a high-speed sequence similarity search tool for proteins predicted from metagenomic reads [24], and further extended it to PZLAST-MAG, enabling similarity searches across the 450 million MAG-derived proteins in Microbiome Datahub [47].

Protein statistics for Microbiome Datahub and MGnify are summarized in Additional File 8. MGnify contains far more protein sequences than Microbiome Datahub, which can be attributed to several factors. First, MGnify incorporates gene prediction results from both FragGeneScan and Prodigal [11]. In addition, MGnify predicts genes from all assembled metagenomic contigs, including those not assigned to binned MAGs, whereas Microbiome Datahub focuses on proteins derived from curated MAG sequences. Furthermore, MGnify and other secondary resources reconstruct MAGs de novo using uniform pipelines, whereas Microbiome Datahub curates original MAGs as reported in the literature. These fundamental differences in underlying data sources and processing strategies are expected to strongly influence protein content and sequence characteristics. Clustering at 90% sequence identity using Linclust [26] yielded 717 million clusters for MGnify and 192 million clusters for Microbiome Datahub. When the two datasets were merged and reclustered, the overlap was limited: 32.9% from the perspective of Microbiome Datahub and 8.8% from MGnify, indicating substantial differences in protein sequence composition between the two resources.

To evaluate similarity at a broader protein family level, we additionally performed clustering at 40% sequence identity [48]. At this threshold, 312 million clusters were obtained for MGnify and 56 million clusters for Microbiome Datahub. Despite the relaxed identity cutoff, the overlap did not increase (29.6% overlap from the Microbiome Datahub perspective and 6.4% from the MGnify perspective). This trend is likely explained, at least in part, by systematic differences in protein length distributions between the databases. In particular, MGnify proteins are on average approximately half the length of proteins in Microbiome Datahub and GlobDB, reflecting the inclusion of shorter gene predictions from fragmented contigs. Because Linclust applies an alignment coverage threshold requiring more than 80% coverage of the sequence pair, many MGnify proteins fail to meet this criterion when compared with longer MAG-derived proteins, thereby reducing apparent overlap even at lower identity thresholds.

To place these observations in a broader genomic context, we downloaded 82,972,511 representative proteins from clusters size >1 in GlobDB release 226 [49] and clustered them with Microbiome Datahub 192 million cluster sequences using Linclust. Clustering at 90% and 40% sequence identity revealed overlaps of 14.8% and 29.8% from the Microbiome Datahub perspective and 34.3% and 52.6% from the GlobDB perspective, respectively. Compared with MGnify, the higher overlap with GlobDB is consistent with more similar protein length distributions and shared MAG-based data sources.

Within Microbiome Datahub, a large fraction of clusters were singletons at both identity thresholds (128 million clusters, 66.8%, at 90% identity; 38 million clusters, 55.2%, at 40% identity), highlighting extensive sequence-level diversity among proteins encoded by MAGs from diverse environments. Together, these results indicate that while sequence-level redundancy across databases is limited, methodological factors, particularly differences in protein length distributions, substantially influence observed overlap, even at relaxed identity thresholds.

To evaluate conservation with isolate genomes, MAG proteins were searched against 12,093,450 RefSeq proteins belonging to MBGD ortholog clusters (with > 2 members), constructed from one isolate genome per genus [9]. On average, 80.7% of proteins per MAG were assigned to MBGD ortholog clusters, indicating substantial overlap between MAG proteins and isolate-derived sequences. However, 72 MAGs had <25% of proteins assigned to MBGD ortholog clusters within the genome, of which 65 belonged to *Patescibacteria*, consistent with previous findings that this lineage harbors a high proportion of novel, lineage-specific genes [50]. In absolute numbers, 625 MAGs contained more than 2000 proteins unassigned to MBGD ortholog clusters. These MAGs were enriched in phyla with large genomes and many uncultured representatives, including *Myxococcota*, *Planctomycetota*, *Asgardarchaeota*, *Acidobacteriota*, and *Cyanobacteriota* (e.g., *Melainabacteria*) (Additional File 9). The environmental distribution of these MAGs is shown in Additional File 10, with the majority derived from activated sludge, followed by lake water, groundwater, and marine sediment, suggesting that these habitats are rich in novel proteins.

**Fig. 5:**
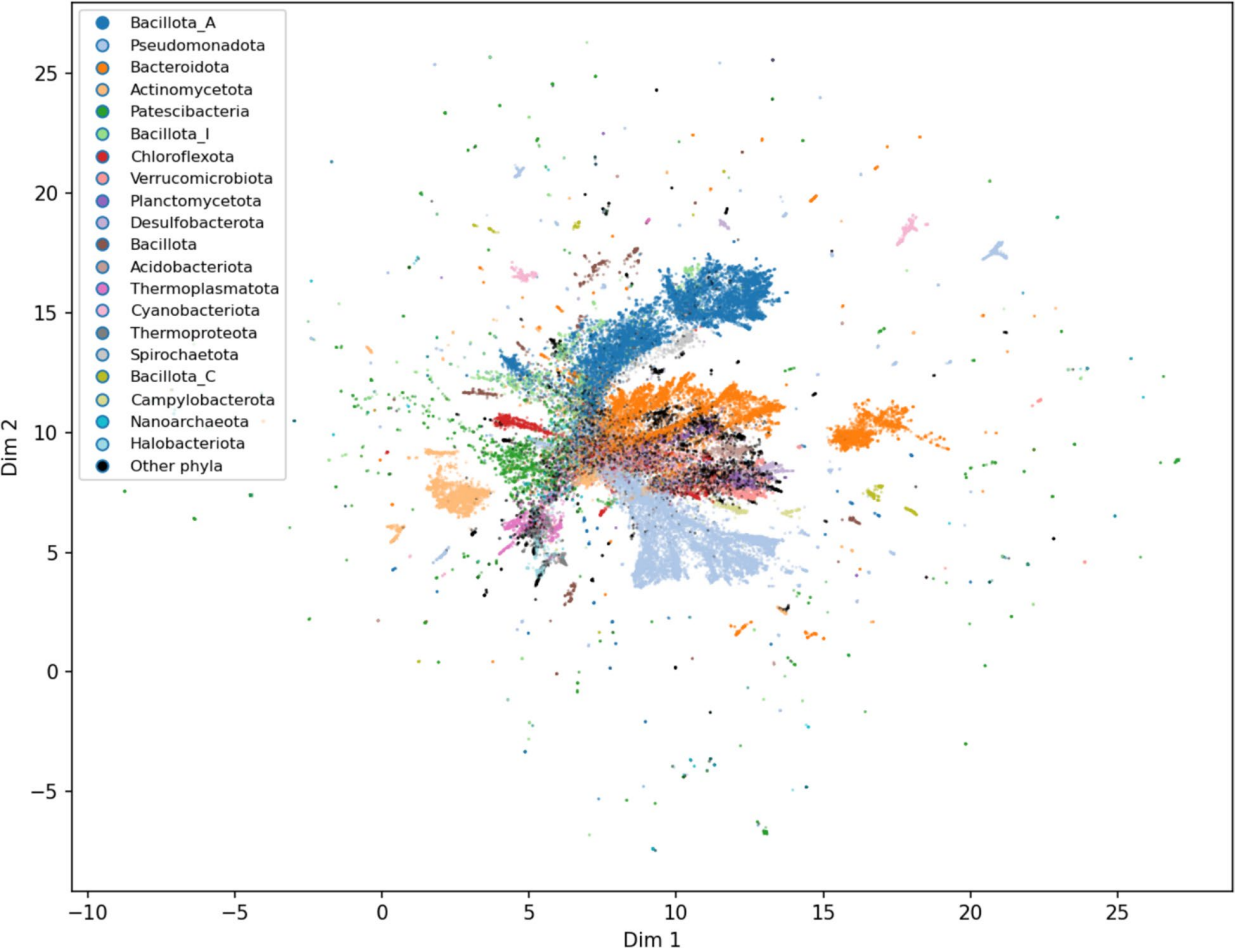
UMAP visualization of KEGG Module composition across the high-quality 172,716 MAGs. The top 20 most abundant phyla are shown in distinct colors, while all other phyla are grouped in black. UMAP was computed using 50 principal components, with a sampling rate of 0.3 for plotting. The analysis and visualization were performed using the Python libraries scikit-learn, umap-learn, and matplotlib

The functional profiles of each MAG were analyzed based on KEGG Module composition, calculated from KO composition using the module completion rate. The KEGG Module compositions of 172,716 high-quality MAGs were visualized in two dimensions using UMAP as an exploratory approach, with the top 15 phyla distinguished by color (Fig. 5). Overall, most MAGs occupied overlapping regions of the UMAP space, reflecting broadly similar core functional modules shared across phyla. Differences among phyla were observed as qualitative trends rather than as strictly separated clusters, and these patterns should be interpreted cautiously, as UMAP is sensitive to parameter choices and is intended for visualization rather than formal clustering. For example, MAGs belonging to *Cyanobacteriota* tended to occupy a distinct region of the UMAP space. Rather than interpreting this pattern as UMAP-driven separation, this trend is consistent with the presence of phylum-specific functional modules, such as those involved in oxygenic photosynthesis, which are largely absent from other bacterial phyla [51].

To further substantiate these observations, we performed a systematic analysis of KEGG Modules enriched in individual phyla (Additional File 11). This analysis identified 39 KEGG Modules that were almost exclusively restricted to a single phylum. Representative examples include oxygenic photosynthesis modules in *Cyanobacteriota* and type II polyketide biosynthesis modules in *Actinomycetota*, both of which are well-established functional hallmarks of these groups [51, 52]. These phylum-specific modules provide an independent functional basis for the qualitative trends observed in the UMAP visualization.

In contrast, the overall correspondence between environmental categories and KEGG Module composition was generally weak, indicating that most functional modules are broadly distributed across multiple environments. However, a targeted enrichment analysis that accounted for phylum-level composition identified a limited number of statistically robust environment-associated signals. Specifically, we detected 19 MEO class–KEGG Module pairs that showed significant enrichment in particular environments after correction for multiple testing and control for phylogenetic bias (Additional File 12). Notably, several of these associations are consistent with established ecological and physiological knowledge. For example, modules related to nitrogen fixation and the cytochrome o ubiquinol oxidase complex were significantly enriched in rhizosphere-associated MAGs, in line with the relatively high oxygen availability and active nitrogen cycling characteristic of root-associated microbial communities [53]. These results indicate that, although strong environment-specific functional signatures are uncommon at the level of KEGG Modules, a small number of biologically interpretable environment–function relationships can nevertheless be detected. The limited number of such associations likely reflects both the broad functional scope of KEGG Modules and the current resolution of environmental annotations.

Both Microbiome Datahub and MGnify contain exceptionally large proteins, with maximum lengths exceeding 57,000 amino acids (Additional File 8). In Microbiome Datahub, 19 proteins larger than 40,000 residues were identified (Additional File 13). As reported previously [54], these proteins originated from the *Omnitrophota* phylum and the *Oscillospiraceae* family within the *Bacillota*_A phylum. Based on the InterProScan search [55], these proteins contain numerous domains within a single polypeptide, and a previous study suggested that such extremely large proteins may be post-translationally processed into multiple smaller proteins [54]. The giant proteins from *Omnitrophota* were identified across multiple orders, differing in both length and sequence, and were primarily recovered from anaerobic environments such as landfills, anaerobic digesters, activated sludge, lake water, and marine sediments. In contrast, the ~40,000-residue proteins from *Oscillospiraceae* were derived from uncultured lineages inhabiting the gut of humans, mice, and rabbits. Cultivation of these lineages and detailed functional analyses of their proteins will be crucial for elucidating the biological roles of giant proteins.

## Discussion

Microbiome Datahub aggregates MAGs from the public sequence repository, auto re-annotates their genes and taxonomy, manually re-annotates environmental metadata using an ontology framework, and infers phenotype information, all of which are provided alongside the sequence data. Re-assembling MAGs using uniform pipelines does not necessarily reproduce the original MAGs reported in individual studies, supporting the motivation for curating and re-annotating original MAGs as implemented in Microbiome Datahub. Microbiome Datahub is designed as a continuously evolving resource rather than a static snapshot of public MAG data. Since the May 2023 release used in this study, the number of MAGs deposited in INSDC has increased substantially and now exceeds the current contents of Microbiome Datahub by more than twofold. To address this, we are actively re-annotating newly released MAGs using the same pipeline described here. The database will be updated as soon as these large-scale computations are completed, and we plan to continue periodic updates thereafter.

### MAG data in public repository

During the development of Microbiome Datahub, we encountered practical challenges related to the reuse of MAG data deposited in public repositories. In the INSDC repositories, metagenomic metadata are primarily recorded in BioSample entries [2]. In practice, nearly all MAGs (>99.9%) are assigned BioSample IDs distinct from those of the original metagenomic read datasets, resulting in metadata being described separately (Additional File 1). This separation is not due to submission errors but is defined in the documentation of INSDC databases (i.e., DDBJ, ENA, and NCBI). In these guidelines, MAG BioSamples are registered under the MIMAG specification, whereas metagenomic read BioSamples are registered under the Minimum Information about a Metagenomic Sequence (MIMS) specification [56], and therefore must be treated as distinct BioSample records. Approximately 35% of MAG-BioSamples could be traced back to the original read BioSample via the derived-from attribute, but the remaining 65% lacked such links, making it difficult to associate MAGs with their source metagenomic samples. Since many studies analyze multiple metagenomes and register multiple MAGs per sample, comparative analyses across MAGs are important. Moreover, MAG reconstruction methods are still developing, and researchers may wish to reassemble MAGs directly from the original read data. Because MAGs alone do not capture the full community composition, linking them to the original read BioSample is also essential for interpreting the ecological niches in microbial communities. To improve data reusability, at a minimum, MAG-BioSample entries should include the BioSample ID of the source metagenome reads using a derived-from or equivalent attribute.

In addition, some MAGs with low assignment rates to MBGD ortholog clusters were found to contain a substantial number of contaminated contigs derived from eukaryotic genomes. Such contamination often does not substantially affect contamination statistics calculated by CheckM, because the presence of large eukaryotic contigs with low gene density inflates genome size but does not strongly alter the copy number of prokaryotic single-copy marker genes used by CheckM. As a result, contamination percentages may be underestimated. This issue is particularly relevant for MAGs derived from host-associated environments or from samples with abundant microbial eukaryotes, where the risk of eukaryotic contamination is high. In such cases, especially when assemblies are highly fragmented, checking genome size and applying additional contamination detection tools is strongly recommended [57].

An important implication of this study is highlighted by our direct comparison between original MAGs deposited in INSDC and MAGs re-assembled from the same BioProject and BioSample by secondary resources such as MGnify and SPIRE (Additional File 5). Although closely related MAGs (often with ANI > 99%) could be recovered across databases, substantial differences were frequently observed in genome contiguity, completeness, genome size, and, in some cases, even whether a corresponding MAG could be recovered at all. These discrepancies demonstrate that differences in assembly and binning strategies alone can lead to divergent MAG representations, even when derived from identical sequencing data.

This finding provides empirical support for an important limitation of secondary MAG databases that rely on uniform re-assembly pipelines: while such approaches offer consistency, they do not necessarily preserve the specific genomic reconstructions originally reported in the literature. In contrast, Microbiome Datahub retains and re-annotates original MAGs as deposited by the submitting authors, thereby preserving genome structures that were evaluated and interpreted in the context of the original studies. This distinction is particularly important for downstream analyses that depend on genome contiguity, gene neighborhood context, or the presence of long and complex genes, as well as for reproducibility and re-evaluation of published findings.

More generally, the quality and consistency of Microbiome Datahub are inherently constrained by the characteristics of the original MAGs deposited in INSDC. In addition to the presence of eukaryotic contamination in some assemblies, older MAGs were often reconstructed using earlier-generation binning algorithms, whereas recent developments (e.g., deep learning/contrastive-learning–based binners) have been shown to recover more high-quality genomes under diverse conditions [58], suggesting that bin quality may be less optimal for some legacy MAG collections compared with MAGs produced using more recent approaches [3]. Furthermore, environmental and sample metadata associated with public submissions are frequently heterogeneous in format, resolution, and completeness, posing substantial challenges for large-scale curation and standardization [2].

Although we applied uniform re-annotation, quality assessment, and metadata harmonization pipelines to mitigate these issues as much as possible, limitations originating from the original submissions inevitably remain. Taken together, Microbiome Datahub should be regarded as a curated and standardized gateway to publicly available MAGs rather than a replacement for careful, study-specific quality control. We therefore encourage users to interpret results derived from individual MAGs with appropriate caution and, where necessary, apply additional filtering or validation steps tailored to their specific research questions.

### MAG environment distribution

It is important to note that the number of MAGs recovered from each environment reflects only their provenance and does not directly represent their true abundance in situ. For example, in Microbiome Datahub, *Prevotella* was the most frequently recovered MAG genus (Fig. 3). However, in the mammalian gut (e.g., humans and mice), genera such as *Bacteroides* are also expected to be highly abundant [59]. The relatively low representation of *Bacteroides* in MAGs is likely explained by several factors: this genus is characterized by high intra-species genomic microdiversity [60], which hampers the generation of long contigs during metagenome assembly and makes MAG reconstruction more difficult. In addition, many *Bacteroides* have recently been reclassified into genera such as *Phocaeicola* and *Parabacteroides* [61]. Similarly, while *Prevotella* is also undergoing taxonomic subdivision (e.g., into *Segatella*) [62], such updates are not yet reflected in GTDB release 220. These combined factors likely contributed to the apparent overrepresentation of *Prevotella* in our dataset.

In general, MAGs can only be assembled when organisms are present above a certain abundance threshold, and high-quality MAGs with high completeness cannot be obtained from extremely rare taxa [3]. Thus, taxa represented by MAGs recovered across many samples in a given environment can be reasonably considered relatively abundant in the environment. At the same time, however, as illustrated by the *Bacteroides* example, biases in MAG reconstructability also influence representation. Therefore, the number of MAGs detected per environment should be interpreted with caution: it serves as a useful indicator of provenance, but not as a direct quantitative measure of abundance.

Moreover, environment–function relationships inferred from MAGs are shaped by the same constraints that affect taxonomic representation. MAG recovery depends on both organismal abundance and genome reconstructability, and thus functional traits encoded by poorly assembled or low-abundance taxa may be underrepresented. In addition, environmental metadata necessarily simplify complex and continuous ecological gradients into discrete categories. As a result, functional adaptations that operate at finer spatial or physicochemical scales may not be fully captured when summarized at the level of MEO classes and KEGG Modules. Importantly, these limitations do not imply that environmental selection is absent, but rather that its genomic signatures are often distributed across combinations of pathways or reflected in regulatory and quantitative differences rather than in the presence or absence of individual modules. Consequently, environment-associated functional signals are expected to be weaker and less frequent than phylogenetically structured ones when assessed using binary module profiles from large-scale MAG datasets.

### MAG protein diversity

Our analysis of protein cluster overlap between Microbiome Datahub and MGnify revealed a low overlap rate of 32.9% (Additional File 8). This low overlap is likely attributable to three main factors: (i) MGnify covers only 18 environmental categories, (ii) MGnify predicts genes not only from binned MAG contigs but also from unbinned or short assembled contigs that are not incorporated into MAGs, and (iii) MGnify employs FragGeneScan for gene prediction [4], a tool optimized for short-read data that tends to over-predict short proteins. Indeed, when we tested FragGeneScan ourselves, it predicted an excessive number of short genes. The imbalance observed in Table 1 between the number of MAGs and proteins in MGnify relative to SPIRE, IMG/M, and Microbiome Datahub, as well as the shorter average protein length, can be explained by the combined effects of FragGeneScan-based gene prediction and the inclusion of genes predicted from short, non-MAG contigs.

Metagenome-derived protein sequences are effective at capturing the diversity of microbial proteins. In particular, MAG-derived proteins are often full-length and thus especially valuable for applications such as structural prediction and evolutionary analyses. This is exemplified by the use of MGnify protein sequences in AlphaFold2/3 [45, 46]. Although Microbiome Datahub contains fewer MAGs and proteins compared with other MAG databases, it encompasses a much broader range of environments and accompanying manual environment annotation. Clustering analyses with MGnify and GlobDB further demonstrate that Microbiome Datahub recovers highly diverse protein sequences (Additional File 8). Notably, approximately 19% of all MAG-derived proteins in Microbiome Datahub could not be assigned to known MBGD ortholog clusters, highlighting their novelty.

## Conclusions

All data in Microbiome Datahub, including MAG genome sequences, predicted gene and protein sequences, genome quality metrics, environmental and taxonomic metadata, ortholog cluster assignments, and phenotype predictions, are accessible via Microbiome Datahub web interface and a data download API. In addition, bulk downloads are available for all MAG genome sequences, protein sequences, 90% and 40% protein cluster representatives, genome statistics, phenotype prediction results, functional module composition matrix, and key metadata including taxonomy and environmental information. These resources collectively establish Microbiome Datahub as a valuable platform that will accelerate future microbiome and microbial genomics research.

## Supplementary Information

Additional file 1: The workflow for MAG metadata extraction from INSDC. MAG metadata in Microbiome Datahub were retrieved from three databases within INSDC.

Additional file 2: Distribution of completeness and contamination in Microbiome Datahub MAGs. Completeness and contamination statistics for each MAG, calculated using CheckM implemented in DFAST_QC.

Additional file 3: Distribution of contig numbers and genome sizes in Microbiome Datahub MAGs. In the MAG contig number histogram, each bin represents 10 contig. Genome size is defined as the sum of the lengths of all contigs in a MAG.

Additional file 4: Distribution of average contig length in Microbiome Datahub MAGs. Each bin represents 1,000 bp. MAGs with an average contig length greater than 200,000 bp are grouped into a single bin.

Additional file 5: Comparative analysis results of original MAGs and re-assembled MAGs from the same BioProject and BioSample across Microbiome Datahub, MGnify, and SPIRE. This table summarizes a targeted comparison of representative original twenty MAGs deposited in INSD and curated in Microbiome Datahub, and corresponding MAGs reconstructed by MGnify and SPIRE from the same BioProject and BioSample. For each MAG, average nucleotide identity (ANI), completeness, contamination, genome size, and contig number are reported. Cases in which no corresponding MAG could be identified in a given database are indicated as N.D.

Additional file 6: Genome size distributions of MAGs from the 30 most represented phyla. Genome size distributions for MAGs from the 30 most abundant MEO environment classes are shown as box plots, with data points also represented in scatter plots.

Additional file 7: Phylum distributions of MAGs across the 20 most common environments. The 20 most abundant phyla are shown in distinct colors. All other phyla are grouped into a single category, shown in gray.

Additional file 8: Protein statistics for Microbiome Datahub, MGnify, and GlobDB. Basic protein statistics for each DB and results of 90% sequence clustering between Microbiome Datahub and the other DBs are shown.

Additional file 9: Phylum distribution of MAGs containing more than 2,000 proteins unassigned to MBGD orthologs. The 20 most abundant phyla containing MAGs with more than 2,000 proteins unassigned to MBGD orthologs are shown.

Additional file 10: Environment distribution of MAGs containing more than 2,000 proteins unassigned to MBGD orthologs. The 10 most abundant MEO environment classes containing MAGs with more than 2,000 proteins unassigned to MBGD orthologs are shown.

Additional file 11: List of 39 KEGG Modules showing strong phylum-level restriction across MAGs. For each KEGG Module, the table reports the number of MAGs having the module within the phylum, the number of MAGs having the module outside the phylum, and the total number of MAGs assigned to the phylum.

Additional file 12: List of 19 KEGG Modules showing statistically significant environment-specific distributions across MAGs after controlling for phylum-level composition. This table summarizes 19 MEO class–KEGG Module pairs showing significant environment-specific enrichment across MAGs after correction for multiple testing and control for phylum-level composition using the Cochran–Mantel–Haenszel test.

Additional file 13: List of 19 proteins longer than 40,000 residues in Microbiome Datahub. MAG ID corresponds to the INSDC genome accession. Environment refers to the environmental annotation summarized from MEO classes and the host NCBI Taxonomy name.

## Data Availability

The Microbiome Datahub data are available through the API at [https://mdatahub.org/apimanual]. Bulk downloads of all MAG sequence data can be accessed at [http://palaeo.nig.ac.jp/Resources/MDatahub/2025/], and the metadata, phenotype prediction results, and functional module composition for the 214,427 MAGs are available at Zenodo 10.5281/zenodo.18073262.

## Abbreviations

API: Application Programming Interface
DB: Database
INSDC: International Nucleotide Sequence Database Collaboration
KEGG: Kyoto Encyclopedia of Genes and Genomes
KO: KEGG Orthology
MAG: Metagenome Assembled Genome
MBGD: Microbial Genome Database for Comparative Analysis
MEO: Metagenome and Microbes Environmental Ontology
MIMAG: Minimum Information about a Metagenome Assembled Genome
MIMS: Minimum Information about a Metagenomic Sequence
UMAP: Uniform Manifold Approximation and Projection
