# Supplementary figures and images for: Microbiome Datahub: an open-access platform integrating environmental metadata, taxonomy, and functional annotation for comprehensive metagenome-assembled genome datasets
Source: Microbiome. 2026 Mar 16;14:119. doi: 10.1186/s40168-026-02385-x (PMC13104272; doi:10.1186/s40168-026-02385-x)

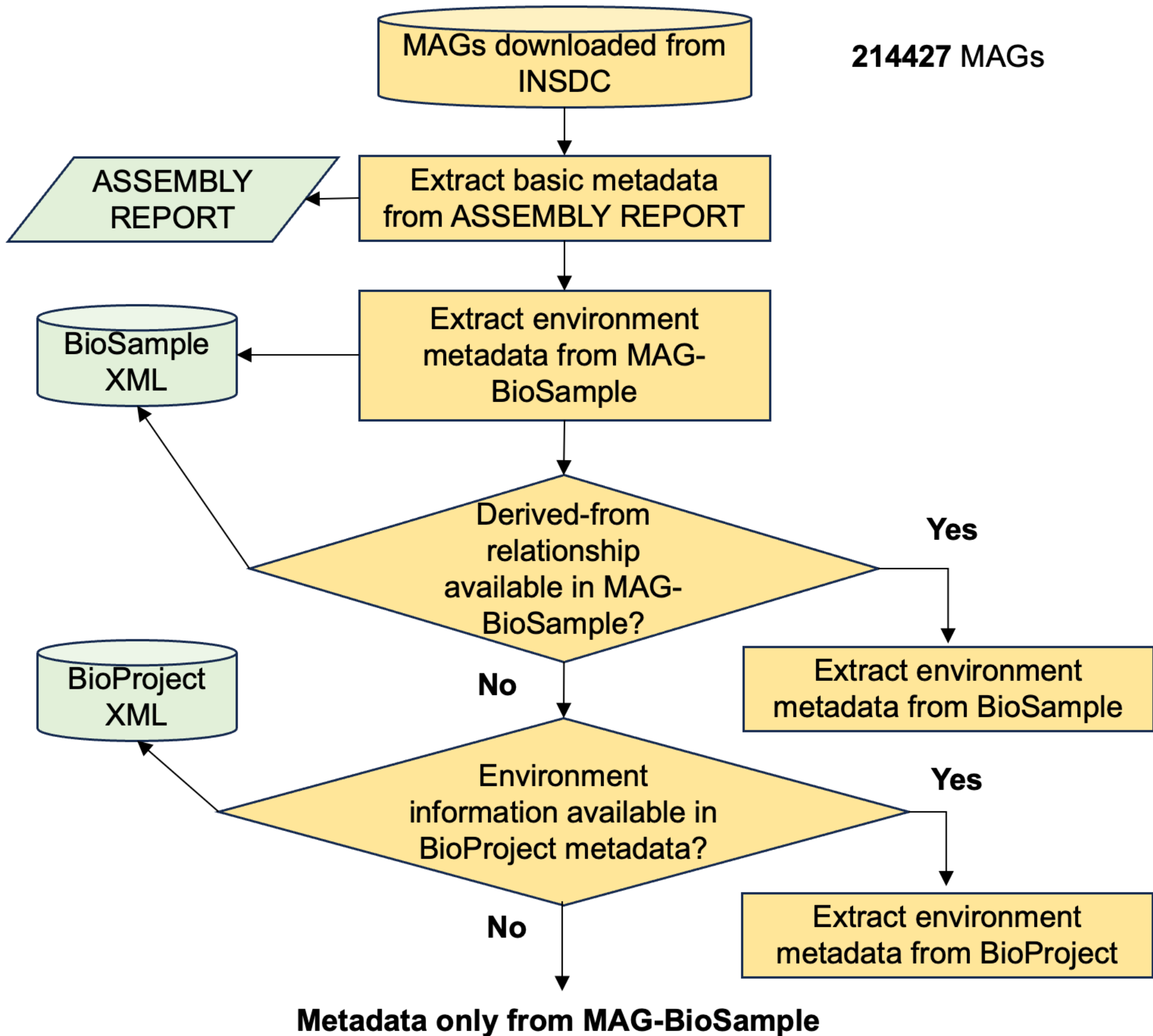

Supplement: Supplementary file 2 — Additional file 1: The workflow for MAG metadata extraction from INSDC. MAG metadata in Microbiome Datahub were retrieved from three databases within INSDC. [file 40168_2026_2385_MOESM1_ESM.pdf]

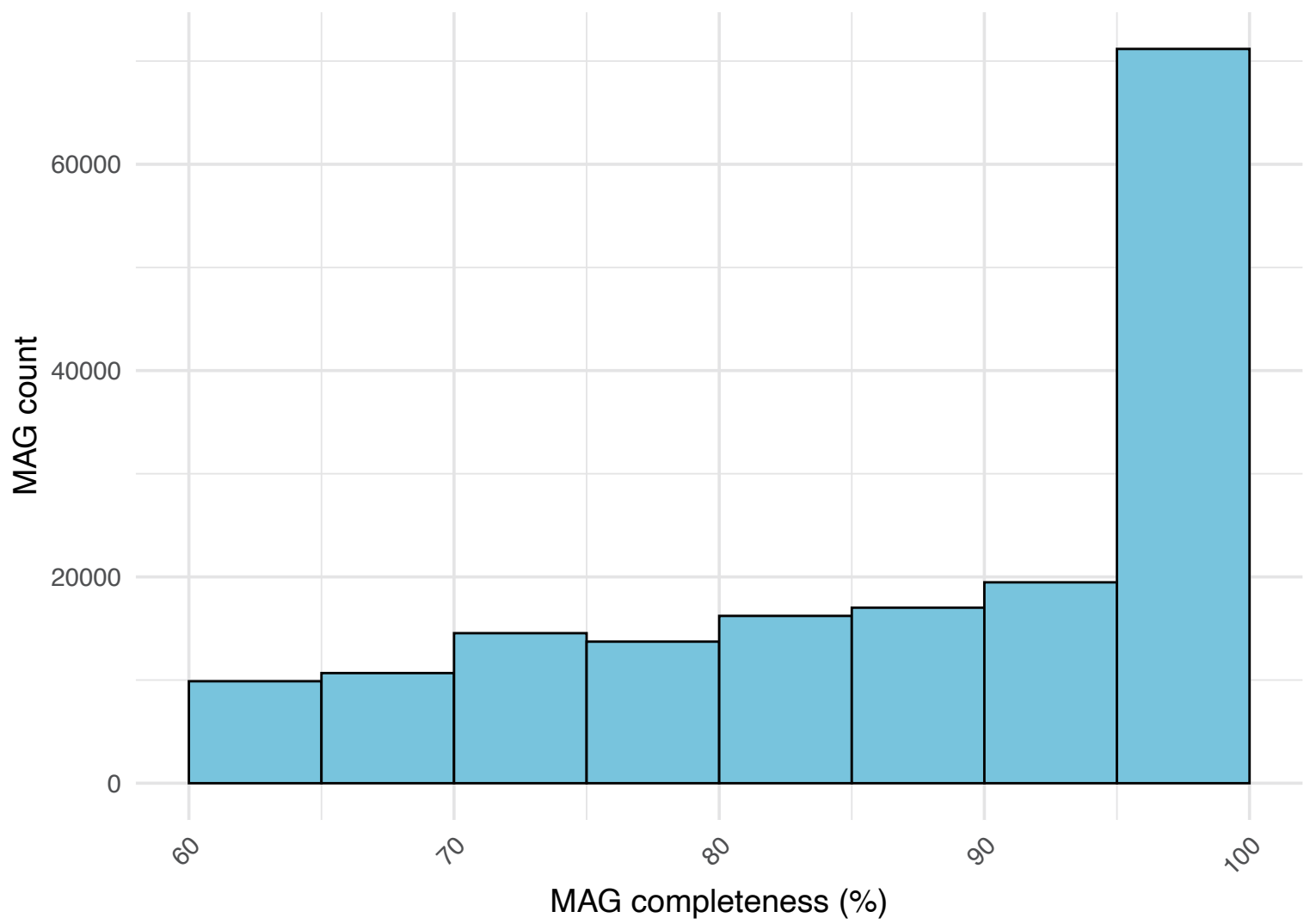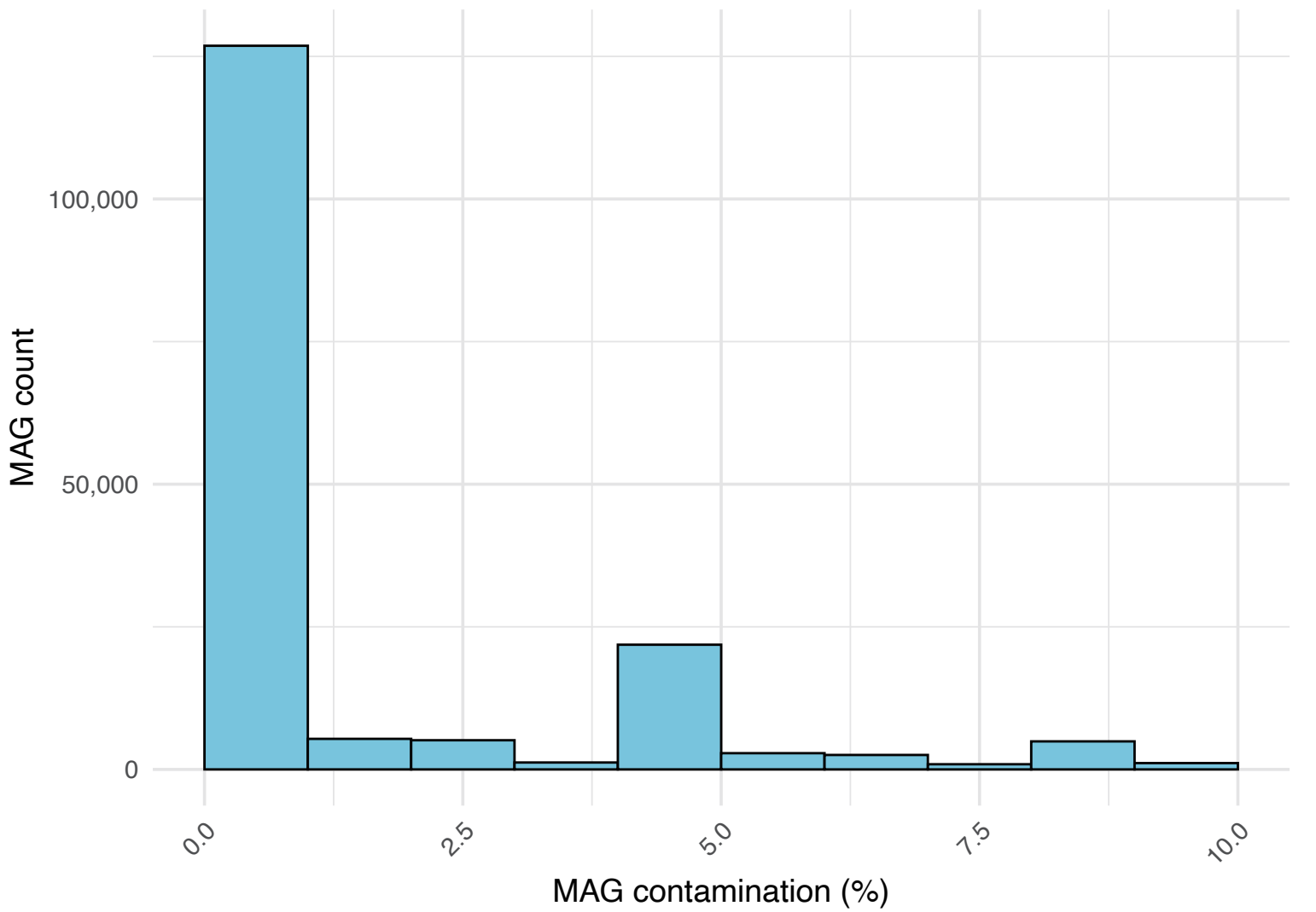

Supplement: Supplementary file 3 — Additional file 2: Distribution of completeness and contamination in Microbiome Datahub MAGs. Completeness and contamination statistics for each MAG, calculated using CheckM implemented in DFAST_QC. [file 40168_2026_2385_MOESM2_ESM.pdf]

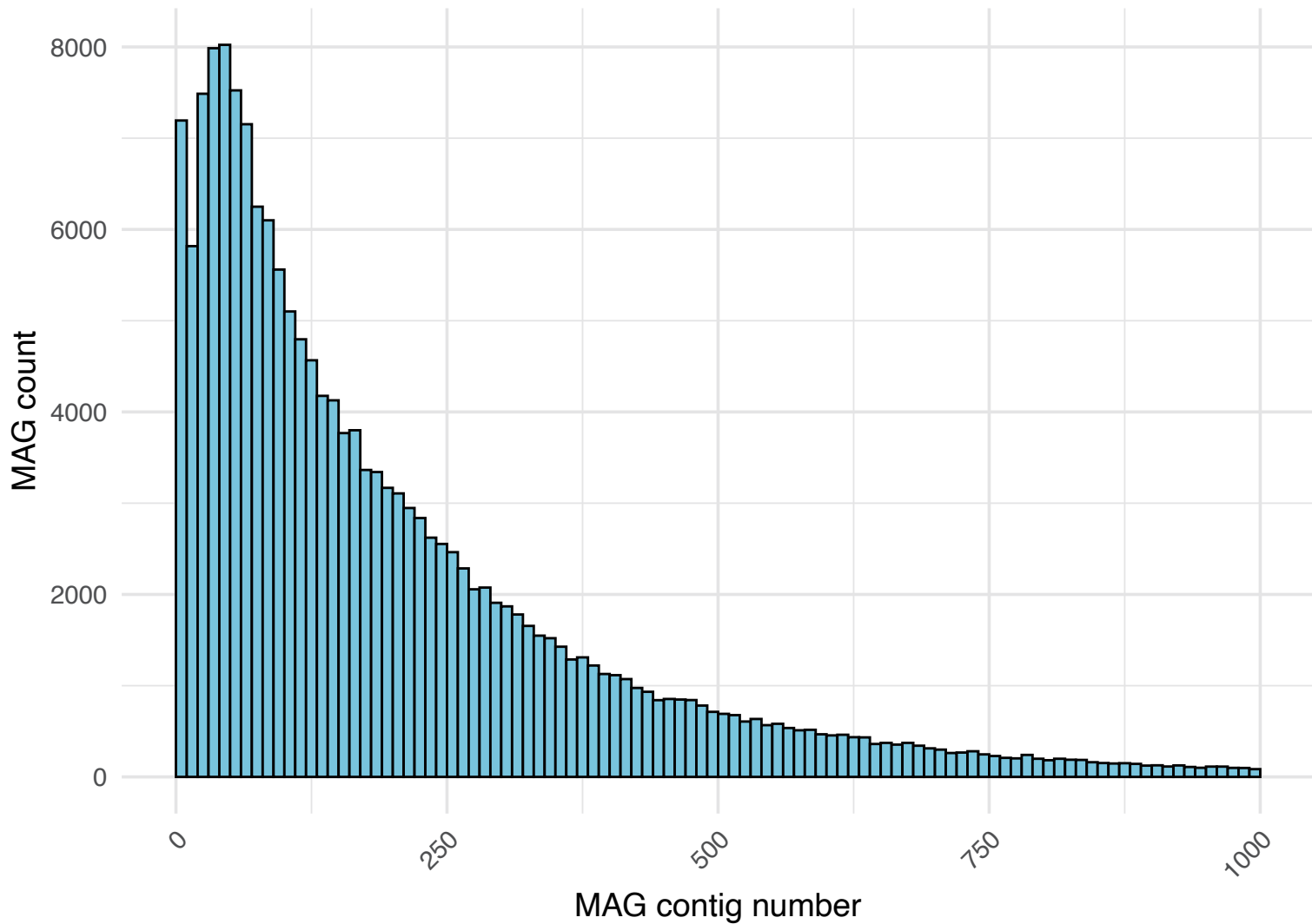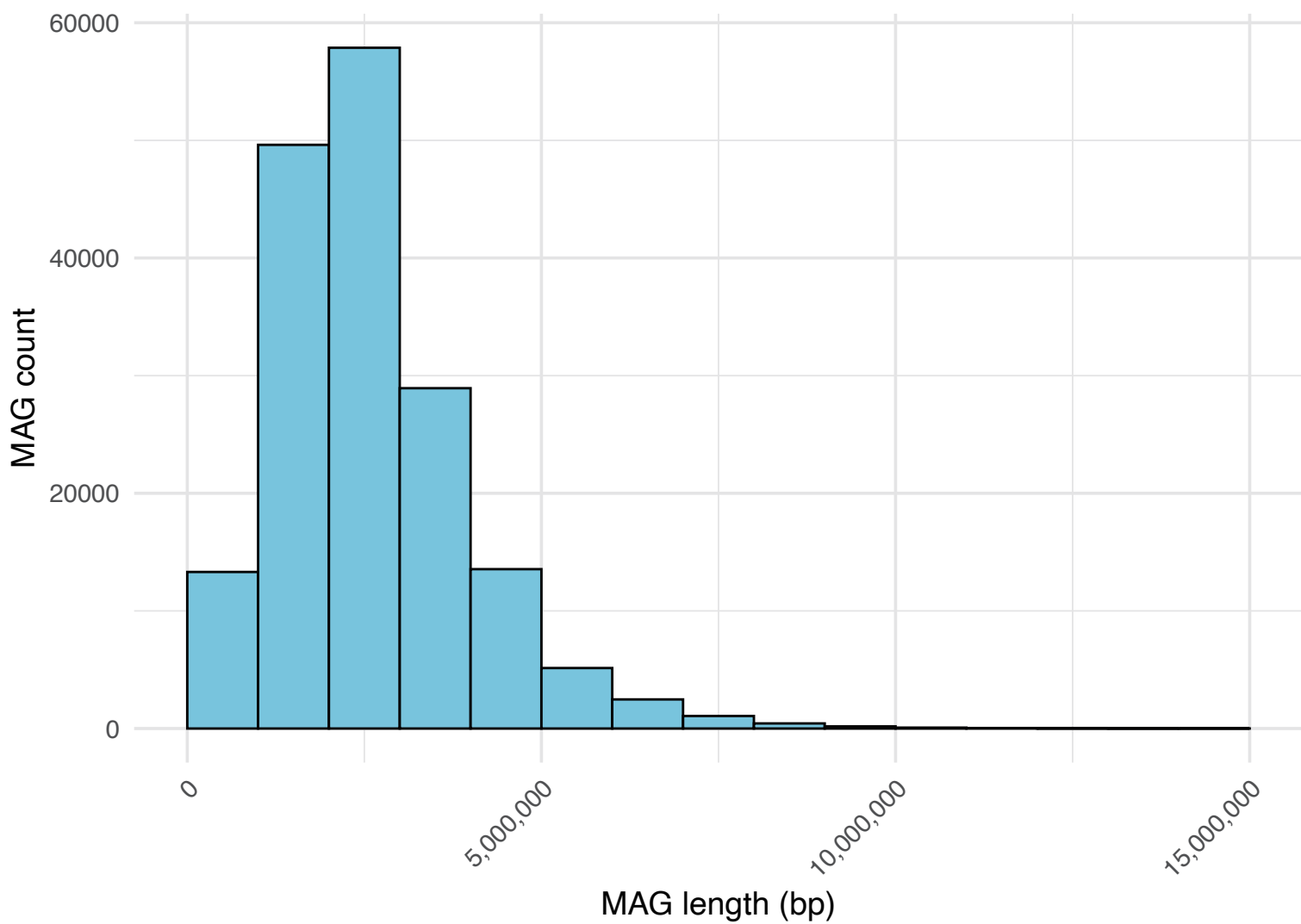

Supplement: Supplementary file 4 — Additional file 3: Distribution of contig numbers and genome sizes in Microbiome Datahub MAGs. In the MAG contig number histogram, each bin represents 10 contig. Genome size is defined as the sum of the lengths of all contigs in a MAG. [file 40168_2026_2385_MOESM3_ESM.pdf]

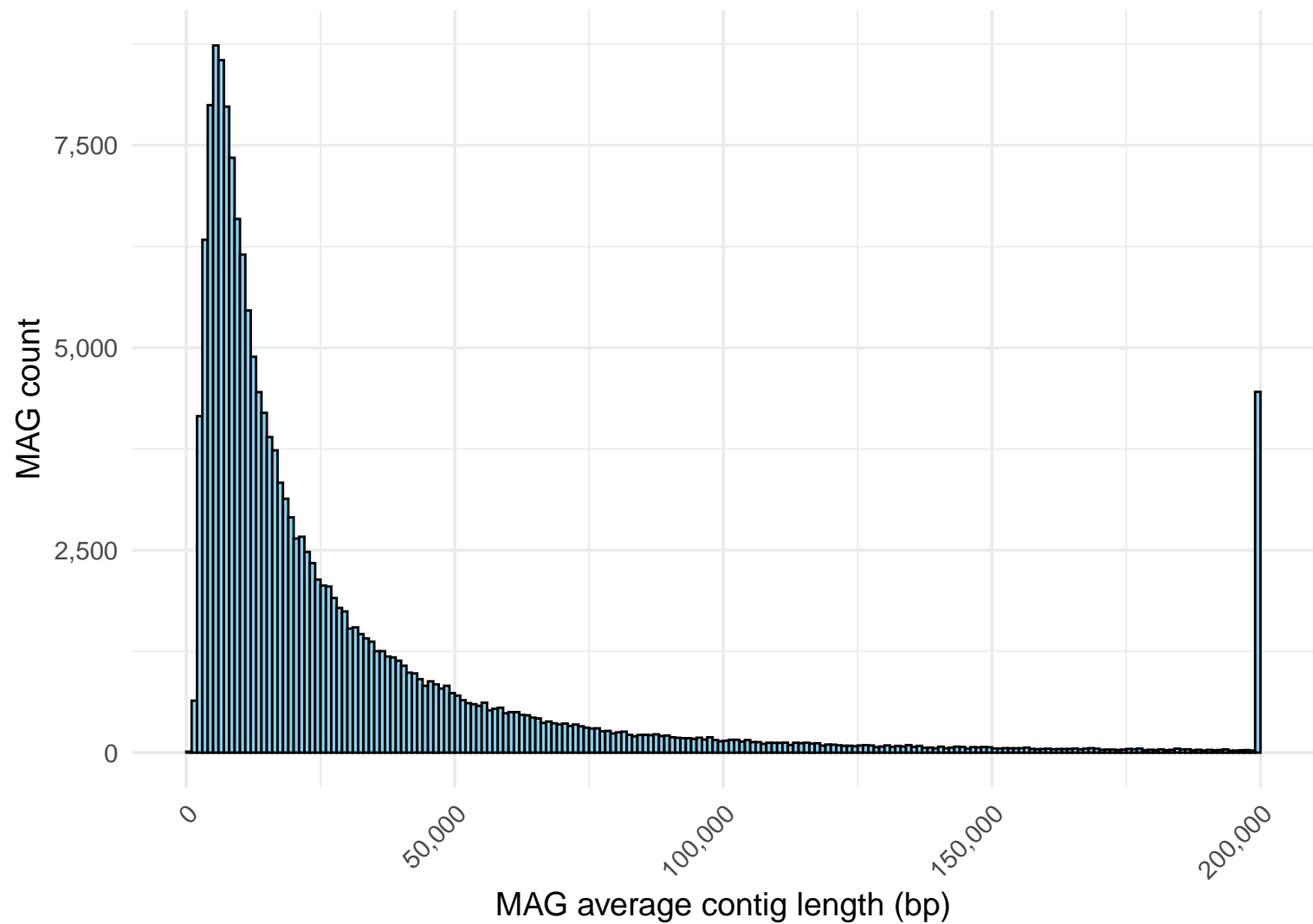

Supplement: Supplementary file 5 — Additional file 4: Distribution of average contig length in Microbiome Datahub MAGs. Each bin represents 1,000 bp. MAGs with an average contig length greater than 200,000 bp are grouped into a single bin. [file 40168_2026_2385_MOESM4_ESM.pdf]

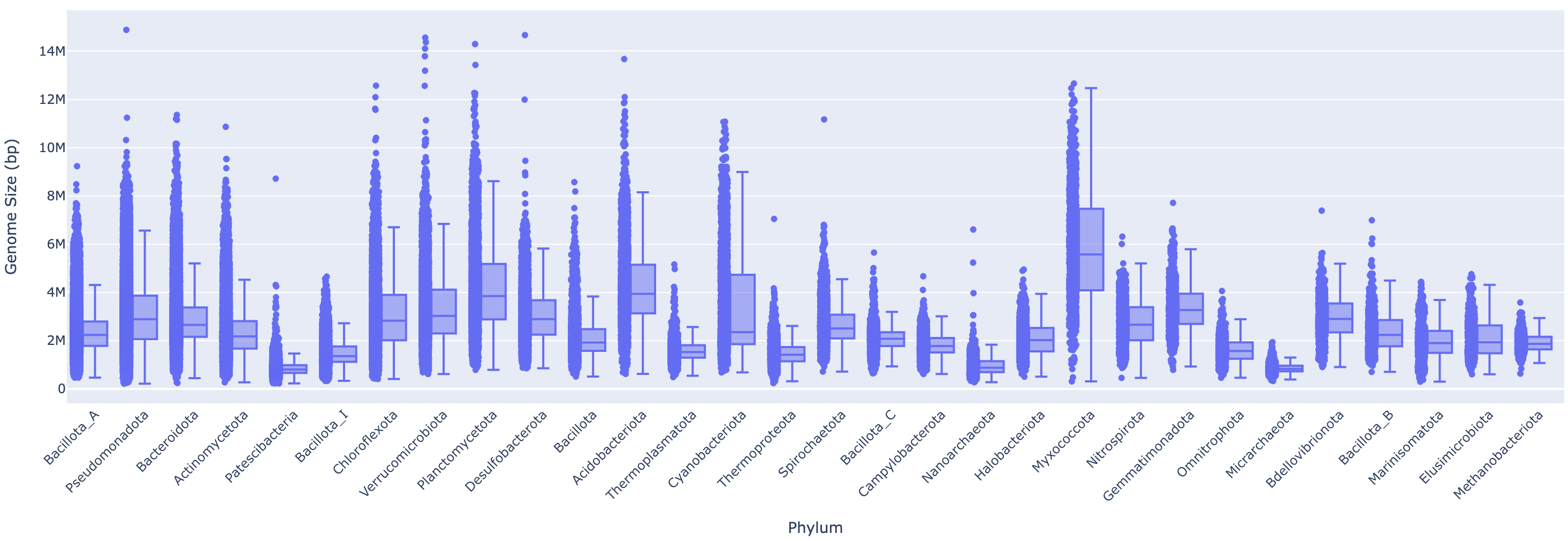

Supplement: Supplementary file 7 — Additional file 6: Genome size distributions of MAGs from the 30 most represented phyla. Genome size distributions for MAGs from the 30 most abundant MEO environment classes are shown as box plots, with data points also represented in scatter plots. [file 40168_2026_2385_MOESM6_ESM.png]

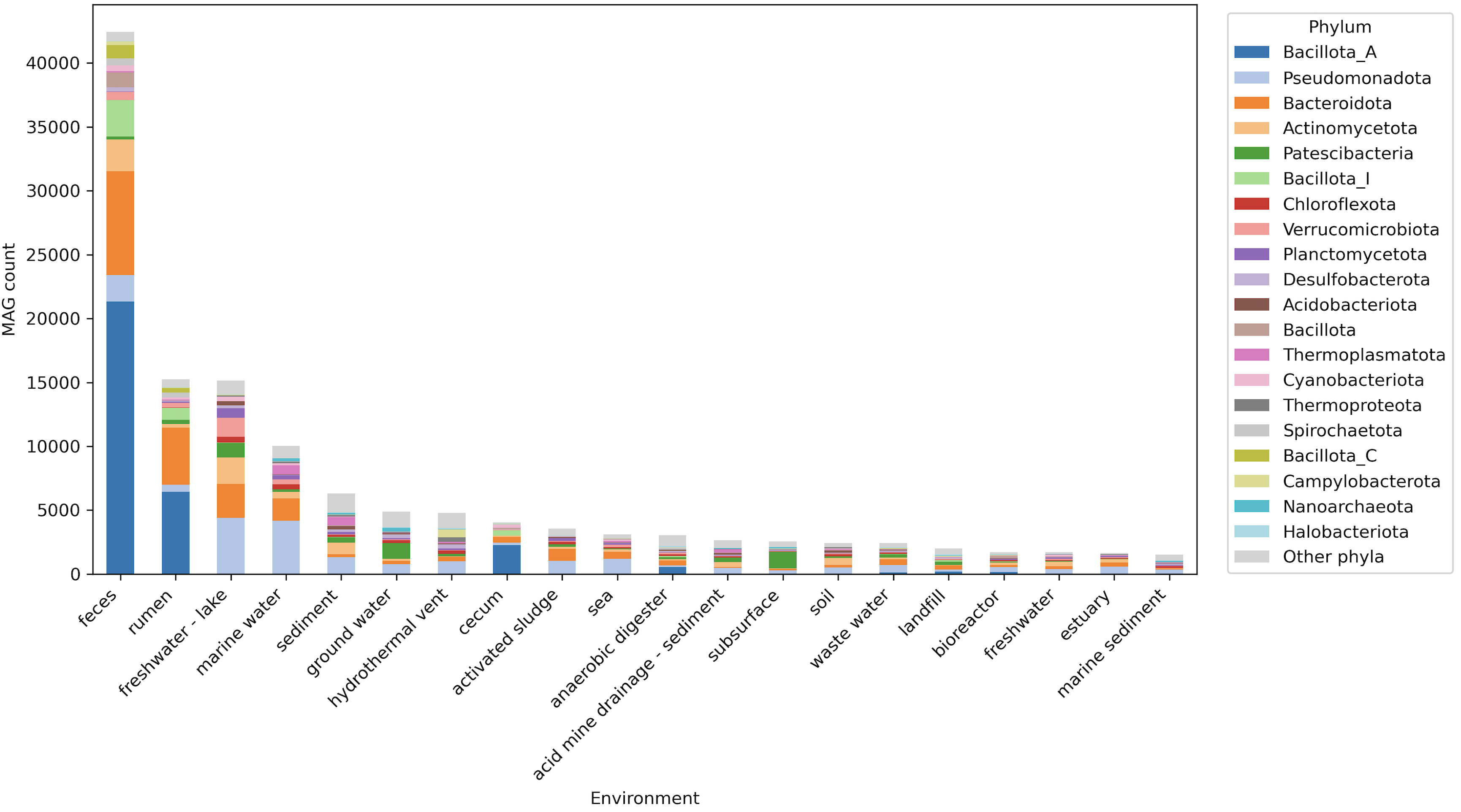

Supplement: Supplementary file 8 — Additional file 7: Phylum distributions of MAGs across the 20 most common environments. The 20 most abundant phyla are shown in distinct colors. All other phyla are grouped into a single category, shown in gray. [file 40168_2026_2385_MOESM7_ESM.png]

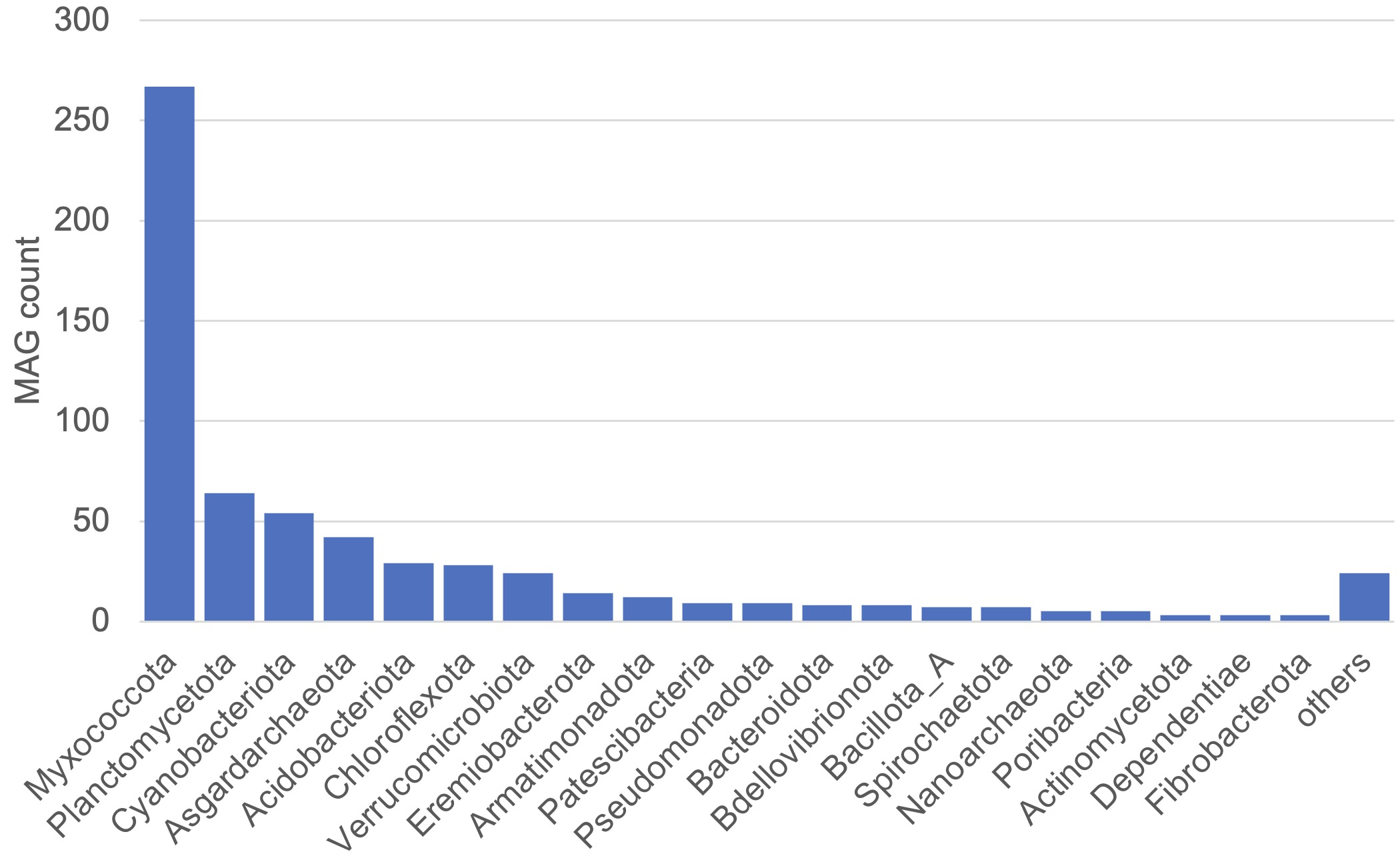

Supplement: Supplementary file 10 — Additional file 9: Phylum distribution of MAGs containing more than 2,000 proteins unassigned to MBGD orthologs. The 20 most abundant phyla containing MAGs with more than 2,000 proteins unassigned to MBGD orthologs are shown. [file 40168_2026_2385_MOESM9_ESM.png]

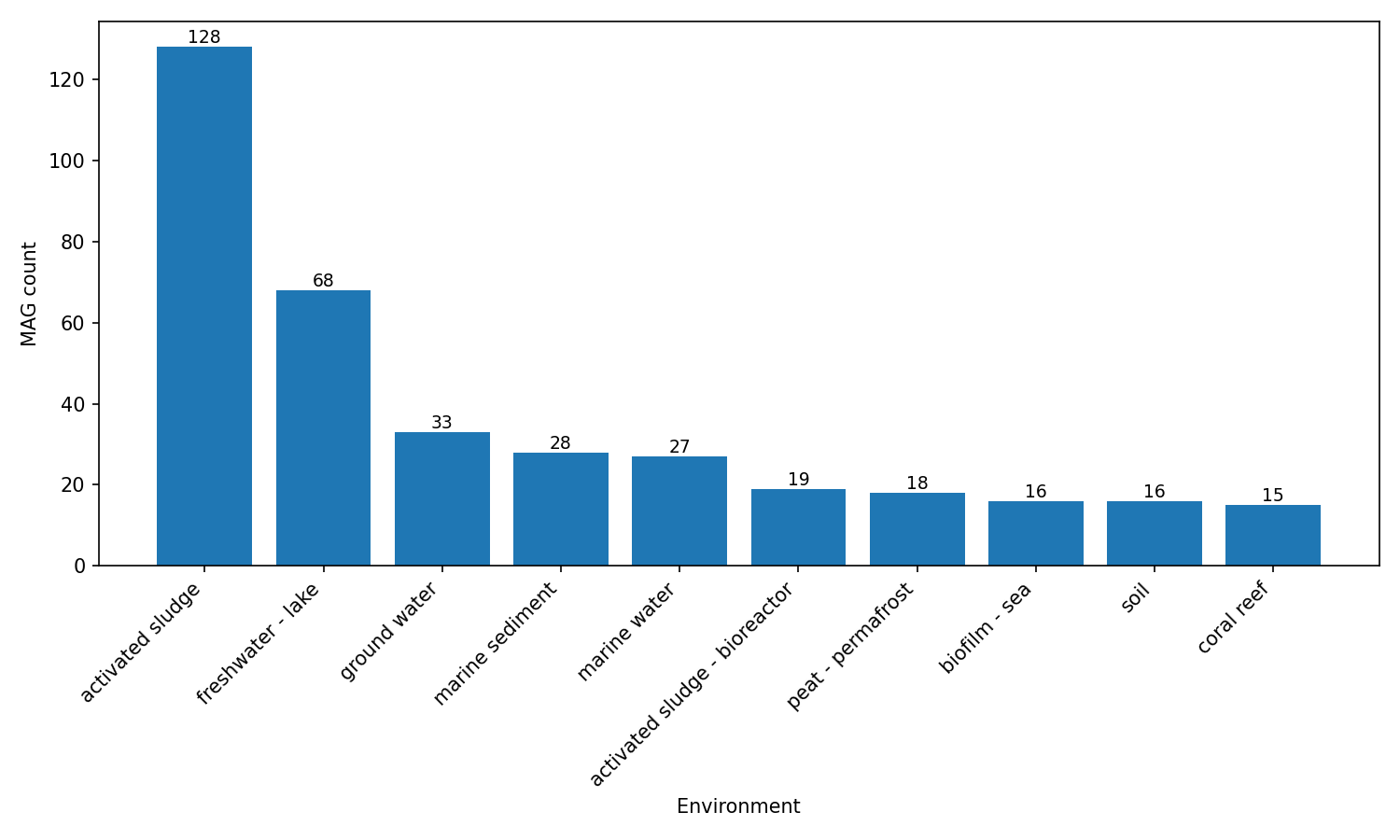

Supplement: Supplementary file 11 — Additional file 10: Environment distribution of MAGs containing more than 2,000 proteins unassigned to MBGD orthologs. The 10 most abundant MEO environment classes containing MAGs with more than 2,000 proteins unassigned to MBGD orthologs are shown. [file 40168_2026_2385_MOESM10_ESM.png]
